# Note to: Hox gene cluster of the ascidian, *Halocynthia roretzi*, reveals multiple ancient steps of cluster disintegration during ascidian evolution

**DOI:** 10.1186/s40851-019-0121-7

**Published:** 2019-02-27

**Authors:** Yuka Sekigami, Takuya Kobayashi, Ai Omi, Koki Nishitsuji, Tetsuro Ikuta, Asao Fujiyama, Noriyuki Satoh, Hidetoshi Saiga

**Affiliations:** 1 Department of Biological Sciences and Technology, Tokyo Metropolitan University, 1-1 Minamiohsawa, Hachiohji, Tokyo 192-0397 Japan; 2 Marine Genomics Unit, Okinawa Institute of Science and Technology Graduate University, Onna, Okinawa, 904-0495 Japan; 3 National Institute of Genetics, 1111 Yata, Mishima, Shizuoka 411-8540 Japan

**Keywords:** Phylogenetic relationship, Tunicates, Ascidians, Hox gene cluster, Disintegration

## Abstract

**Background:**

In the previous paper published in 2017, we described the structure of Hox gene cluster of the ascidian, *Halocynthia roretzi*, and discussed the scenario for the disintegration of Hox gene clusters during evolution of ascidians. The description about the Hox gene cluster structure still represents the latest information, hence it has been left unchanged. In contrast, some points in Discussion, the description on the phylogenetic relationships among tunicates and the theoretical scenario for the disintegration of Hox gene cluster during evolution of ascidians, should be changed because the phylogenetic relationships among tunicates have recently been updated. The above mentioned points were made in accordance with the phylogenetic tree for tunicates based on the mitochondrial DNA sequences, which was the latest at the time of publication. In 2018, however, Kocot et al. and Delsuc et al. proposed new phylogenetic trees for tunicates based on a large number of nuclear gene sequences. The trees obtained by the two groups are essentially the same and different from the previous one in the phylogenetic positions of Appendicularia and Thaliacea, which leads to a change in the order of the emergence of ascidians and the Hox gene cluster disintegration during evolution of ascidians or tunicates.

**Results:**

We add here a note to update the previous description on the phylogenetic relationships among tunicates and the theoretical scenario, including one Figure, so as to coincide with the new phylogenetic relationships among tunicates based on the nuclear gene sequences.

**Conclusion:**

The previous summarized conclusion remains unchanged: we suggest that the Hox gene cluster of the ancestral ascidian experienced extensive genome shuffling during the course of evolution to *Hr* and *Ci*. Nevertheless, some features are shared in Hox gene components and gene organization on the chromosomes, suggesting that Hox gene cluster disintegration in ascidians involved early events common to all ascidians and later lineage-specific events.

**Electronic supplementary material:**

The online version of this article (10.1186/s40851-019-0121-7) contains supplementary material, which is available to authorized users.

## Background

No change; identical to the previously published version.

## Materials and methods

No change; identical to the previously published version.

## Results

No change; identical to the previously published version.

## Discussion

Following changes should be made:

Pages 7–8 of Sekigami et al. Zoological Letters (2017) 3:17

DOI 10.1186/s40851-017-0078-3: The second sentence in the beginning of Discussion should be replaced as follows:

*Tunicates are divided into three branches; one comprises Appendicularia, another comprises Stolidobranchia (including Hr) and the other consists of Phlebobranchia (including Ci), Aplousobranchia (another ascidian group) and Thaliacea [33, 34]*:

Pages 9–10 of Sekigami et al. Zoological Letters (2017) 3:17

DOI 10.1186/s40851-017-0078-3: The second paragraph of “Conclusion: a theoretical scenario for the disintegration of the Hox gene cluster in the ascidian or tunicate evolution” should be replaced as follows: (please find the change from the previously published version in italics):

In this scheme, 1) when the ancestral chordate emerged, it had a single Hox gene cluster consisting of three anterior (PGs 1–3), five central (PGs 4–8) and three ancestral posterior (PG9/10, PG11/12 and PG13/14) genes [30]. 2) The ancestral chordate evolved, and the last common ancestor of tunicates and vertebrates diverged from the lineage to cephalochordate. *3) When the ancestral tunicate diverged from the lineage to vertebrates, it must have experienced extensive genomic rearrangement, including the loss of at least one (or two) central Hox genes and early disintegration events in the Hox gene cluster, and likely came to possess tunicate characteristics. The loss of the central Hox genes and disintegration of the Hox gene cluster may be correlated with peculiar way of development of tunicates [12] and/or limited function of Hox genes as observed in the early development of Ci [32]. 4) The ancestral tunicate evolved, being diverged from the lineage to Appendicularia, into a common ancestor of Ascidiacea and Thaliacea, and the Hox gene complement consisting of nine genes (three each of anterior, central, and posterior Hox genes) was established. 5) The ancestor, in turn, evolved into two lineages; one giving rise to Pleurogona ascidians (Stolidobranchia) and the other to Thaliacea and Enterogona ascidians (Phlebobranchia and Aplousobranchia). During evolution giving rise to Hr and Ci, the Hox gene cluster as well as the genome must have experienced further genomic rearrangement in different manners.* The relatively small conserved gene arrangement between *Hr* and *Ci* in the regions surrounding Hox genes may support this part of the scenario.

**Fig. 5R:**
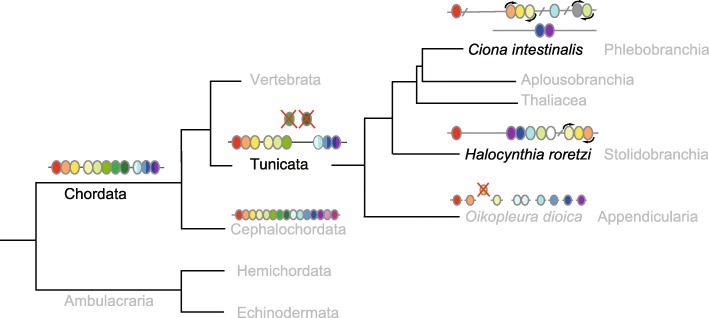
A proposed scheme for Hox gene cluster disintegration during ascidian evolution. The last common ancestor for cephalochordates, tunicates, and vertebrates (represented as Chordata) possessed a single Hox gene cluster consisting of three anterior (red, orange, and yellow), five central (green) and three ancestral posterior genes (blue). After the ancestral cephalochordate diverged, the tunicate ancestor (represented as Tunicata), in turn, diverged from the vertebrate lineage. At this stage, the ancestral tunicate must have experienced extensive changes in the genome, and the Hox gene cluster disintegration started, losing one or two central Hox genes. *The ancestral tunicate subsequently evolved, being separated from Appendicularia, into two lineages; one leading to stolidobranchian ascidians (Hr) (right side, middle) and the other leading to phlebobranchian (Ci) and aplousobranchian ascidians as well as Thaliacea (right side, upper) [33, 34]. By the divergence of the two evolutionary lineages, the Hox gene complement of the ancestral ascidian with each three of anterior, central and posterior genes must have been established. At the same time, early Hox gene cluster disintegration events must have occurred. Then, the Hox gene cluster further disintegrated in different patterns in the evolutionary lineages giving rise to Hr and Ci.* White or gray ovals indicate Hox genes, probably of the central Hox gene group origin (see text). The Hox gene complement of *Oikopleura dioica*, consisting of two anterior, one central, and six posterior genes, and that of amphioxus, consisting of 15 members, are schematically represented.

## Declarations

No change; identical to the previously published version.

## References

Page 11 of Sekigami et al. Zoological Letters (2017) 3:17

DOI 10.1186/s40851-017-0078-3: The following two references should be added to the previously published version:

33 Kocot KM, Tassia MG, Halanych KM, Swalla BJ. Molecular phylogenetics and evolution phylogenomics offers resolution of major tunicate relationships. Mol. Phylogenet. Evol. 2018;121:166–73. 10.1016/j.ympev.2018.01.005

34 Delsuc F, Philippe H, Tsagkogeorga G, Simion P, Tilak M, Turon X, et al. A phylogenomic framework and timescale for comparative studies of tunicates. BMC Biology. 2018;16:39. https://doi.org/10.1186/s12915-018-0499-2.

## Legends for Figures

No change for Figs. 1–4, s1-s3; identical to the previously published version.

Page 9 of Sekigami et al. Zoological Letters (2017) 3:17

DOI 10.1186/s40851-017-0078-3: Fig. 5 legend please find the change from the previously published version in italics.

Page 9 of Sekigami et al. Zoological Letters (2017) 3:17

DOI 10.1186/s40851-017-0078-3: Fig. 5 should be replaced by Fig. 5R in a separate sheet.

## Additional file

Additional file 1: Figure 5R.

