# Supplementary figures and images for: Note to: Hox gene cluster of the ascidian, Halocynthia roretzi, reveals multiple ancient steps of cluster disintegration during ascidian evolution
Source: Zoological Lett. 2019 Feb 27;5:8. doi: 10.1186/s40851-019-0121-7 (PMC6394070; doi:10.1186/s40851-019-0121-7)

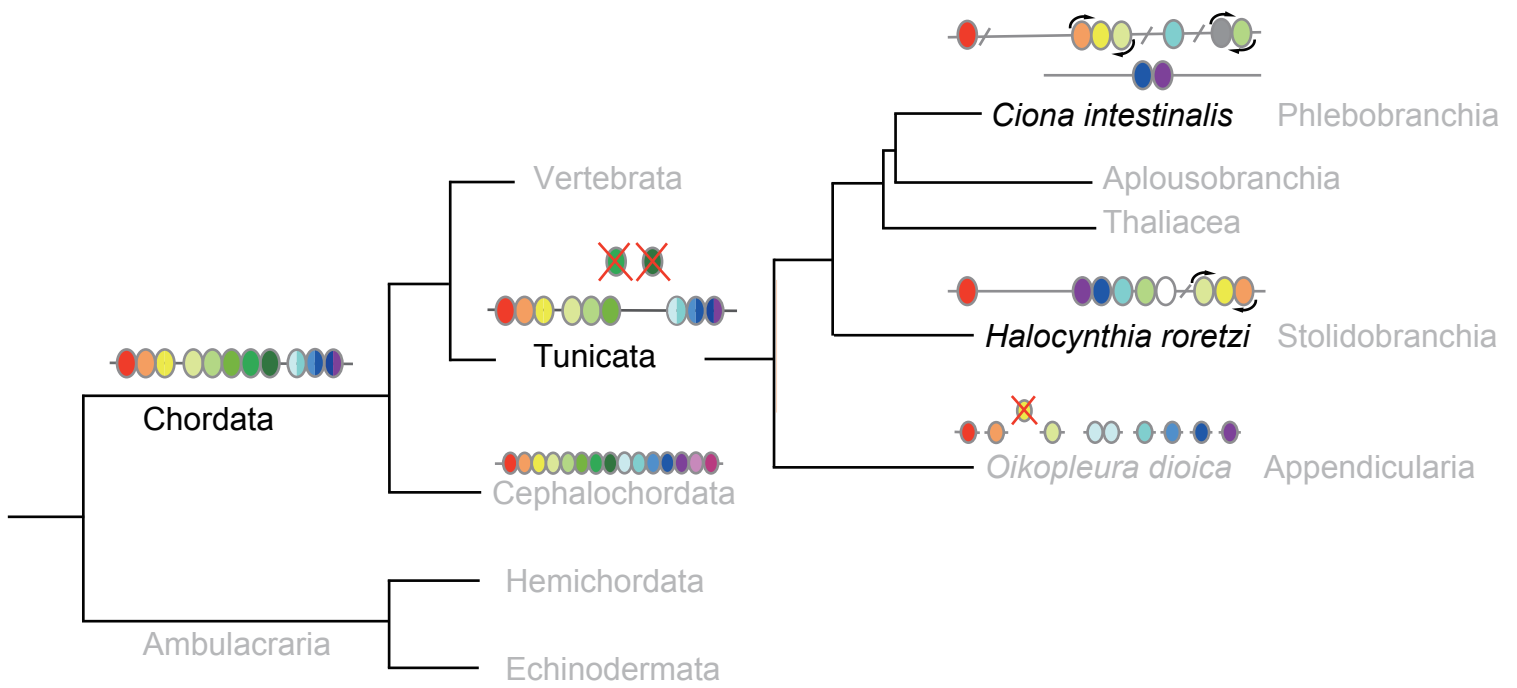

Supplement: Supplementary file 1 — Figure 5R. [file 40851_2019_121_MOESM1_ESM.pdf]
